# Supplementary material for: Recovery-induced tipping in Stommel’s kicked ocean box model
Source: PLoS One. 2026 Feb 3;21(2):e0342171. doi: 10.1371/journal.pone.0342171 (PMC12867227; doi:10.1371/journal.pone.0342171)
Supplement: S1 File — This document supplies further details on the reduction from Eq (1) to Eq (3). (PDF) [file pone.0342171.s001.pdf]

S1 File. Stommel model reduction. This file supplies further details on the reduction from Eq (1) to Eq (3).

The exposition in [1] describes the same general process but reverses indexing on the two boxes. To avoid confusion, we summarize the reduction process using Stommel's original box indexing choice, which we follow.

By choosing temperature and salinity scales such that $T_2^* = -T_1^*$ and $S_2^* = -S_1^*$, one finds that the average temperature $\bar{T} = \frac{1}{2}(T_1 + T_2)$ satisfies

$$\frac{d\bar{T}}{dt} = -c\bar{T}$$

while the average salinity $\bar{S} = \frac{1}{2}(S_1 + S_2)$ satisfies

$$\frac{d\bar{S}}{dt} = -d\bar{S}.$$

The average temperature and salinity in the two-box system thus decays towards zero. To study the long-term behavior of the system it suffices to track the difference between the two boxes using state variables $\Delta T = T_1 - T_2$ and $\Delta S = S_1 - S_2$. By combining Eq (1a) with Eq (1b) and Eq (1c) with Eq (1d) we obtain

$$\frac{d\Delta T}{dt} = c(\Delta T^* - \Delta T) - 2|q|\Delta T \qquad \text{(S1.1a)}$$

$$\frac{d\Delta S}{dt} = d(\Delta S^* - \Delta S) - 2|q|\Delta S \qquad \text{(S1.1b)}$$

where $\Delta T^* = T_1^* - T_2^* = 2T_1^*$ and $\Delta S^* = S_1^* - S_2^* = 2S_1^*$. Further, the circulation given in Eq (2) simplifies to

$$q = k(\beta\Delta S - \alpha\Delta T). \qquad \text{(S1.2)}$$

Finally, the change of variables $x = \Delta S/\Delta S^*$, $y = \Delta T/\Delta T^*$, and $\tau = ct$ renders Eqs (S1.1) in the dimensionless form

$$\frac{dx}{d\tau} = \delta(1 - x) - |f|x \qquad \text{(S1.3a)}$$

$$\frac{dy}{d\tau} = 1 - y - |f|y \qquad \text{(S1.3b)}$$

where $\delta = d/c$, and Eq (S1.2) reduces to nondimensional circulation

$$f = \frac{1}{\lambda}(Rx - y), \tag{S1.4}$$

with $\lambda = c/(2\alpha k \Delta T^*)$ and $R = \beta \Delta S^*/(\alpha \Delta T^*)$. Substituting Eq (S1.4) into Eqs (S1.3a,b) yields the nondimensional system given in Eqs (3).

References

1. Kaper H, Engler H. Mathematics and Climate. SIAM; 2013.
