# Supplementary material for: Recovery-induced tipping in Stommel’s kicked ocean box model
Source: PLoS One. 2026 Feb 3;21(2):e0342171. doi: 10.1371/journal.pone.0342171 (PMC12867227; doi:10.1371/journal.pone.0342171)
Supplement: S2 File — This document presents rough estimates of kicks and flow times corresponding to Great Salinity Anomalies in the North Atlantic and annual freshwater input from Greenland. (PDF) [file pone.0342171.s002.pdf]

S2 File. Estimating kicks and flow times from geophysical data. This document presents rough estimates of kicks and flow times corresponding to Great Salinity Anomalies in the North Atlantic and annual freshwater input from Greenland.

Great Salinity Anomalies

Estimates of salinity drops associated with Great Salinity Anomalies in the North Atlantic come from Figure 1c in [1], Figure 4e in [2], and Figure 5 in [3]. We convert the estimated variability of 0.1 to 0.5 practical salinity units (PSU) to the nondimensional salinity variable x in Stommel's model as follows. Two different North Atlantic salinity measurements S_2 and $\hat{S}_2$ correspond to nondimensional salinities

$$x = \frac{S_1 - S_2}{S_1^* - S_2^*} \quad \text{and} \quad \hat{x} = \frac{S_1 - \hat{S}_2}{S_1^* - S_2^*}$$

whose difference is

$$\hat{x} - x = -\frac{\hat{S}_2 - S_2}{S_1^* - S_2^*}. \tag{S2.1}$$

From [1–3] we have $\hat{S}_2 - S_2 \in [-0.5, -0.1]$ PSU. We estimate $S_1^* - S_2^*$, the difference in bath salinities, using data from the World Ocean Atlas from 2015-2022: ocean surface salinities averaged about 34.5–35 PSU in the North Atlantic and achieved maximum averages of 37–37.5 PSU at lower latitudes in the Atlantic [4]. The local forcing salinities S_1^* and S_2^* should be more extreme than these observed values, so we consider $S_1^* \in [38, 39]$ PSU and $S_2^* \in [33, 34]$ PSU. This yields the estimate $S_1^* - S_2^* \in [4, 6]$ PSU. Matching extremes from the estimates of $\hat{S}_2 - S_2$ and of $S_1^* - S_2^*$ we obtain lower and higher estimates

$$(\hat{x} - x)_{\text{low}} = -\frac{-0.1 \text{ PSU}}{6 \text{ PSU}} \approx 0.02$$
$$(\hat{x} - x)_{\text{high}} = -\frac{-0.5 \text{ PSU}}{4 \text{ PSU}} \approx 0.1$$

to one significant figure. We interpret these as salinity kicks in the range $0.02 \leq \kappa \leq 0.1$.

From Figure 1c in [1], we estimate time periods between Great Salinity Anomalies at 7–22 years. Using the nondimensionalization $\tau = ct$ (see S1 File) and $c = 1/25$

days^{-1} [5], we obtain

$$\tau_{\min} = \frac{1}{25 \text{ days}} \cdot \frac{365 \text{ days}}{1 \text{ year}} \cdot 7 \text{ years} \approx 100$$
$$\text{and} \quad \tau_{\max} = \frac{1}{25 \text{ days}} \cdot \frac{365 \text{ days}}{1 \text{ year}} \cdot 22 \text{ years} \approx 320.$$

Meltwater input from Greenland

We estimate salinity changes associated with yearly freshwater input from Greenland into the Irminger Sea as follows. Let $c_{1,2}$ and $v_{1,2}$ be the salinity and volume, respectively, of the Irminger Sea before ($_1$) and after ($_2$) freshwater input. Further, let $\Delta v = v_2 - v_1$ represent the volume of freshwater input. Combining the formulae

$$c_1 v_1 = c_2 v_2 \quad \text{and} \quad v_2 = v_1 + \Delta v$$

we have

$$c_2 = \frac{c_1 v_1}{v_1 + \Delta v}.$$

Using an area of $780,000$ km^2 for the Irminger Sea and an upper layer depth of 0.1 km, we estimate a surface volume $v_1 = 78,000$ km^3. Further, the salinity in the upper layer of the Irminger Sea is approximately $c_1 = 35$ PSU [1]. Using $\Delta v = 100$ km^3 yields

$$c_2 = \frac{(35 \text{ PSU})(78,000 \text{ km}^3)}{78,000 \text{ km}^3 + 100 \text{ km}^3} \approx 34.9551856594 \text{ PSU}$$

while using $\Delta v = 350$ km^3 yields

$$c_2 = \frac{(35 \text{ PSU})(78,000 \text{ km}^3)}{78,000 \text{ km}^3 + 350 \text{ km}^3} \approx 34.8436502872 \text{ PSU}.$$

These values of c_2 correspond to a change in salinity $c_2 - c_1 \equiv \hat{S}_2 - S_2$ in the range of -0.045 to -0.16 PSU. Normalizing this by the difference $S_1^* - S_2^* \in [4, 6]$ PSU between surrounding basins (see Eq S2.1), we obtain lower and upper estimates

$$(\hat{x} - x)_{\text{low}} = -\frac{-0.045}{6} \approx 0.007$$

and

$$(\hat{x} - x)_{\text{high}} = -\frac{-0.16}{4} = 0.04.$$

To convert an annual return period to nondimensional τ, we use $\tau = ct$ (see S1 File) and $c = 1/25\ \text{days}^{-1}$ [5]:

$$\tau = \frac{1}{25\ \text{days}} \cdot \frac{365\ \text{days}}{1\ \text{year}} \approx 15.$$

References

1. Biló T, Straneo F, Holte J, Le Bras IA. Arrival of new great salinity anomaly weakens convection in the Irminger Sea. Geophysical Research Letters. 2022;49(11):e2022GL098857.

2. Holliday NP, Bersch M, Berx B, Chafik L, Cunningham S, Florindo-López C, et al. Ocean circulation causes the largest freshening event for 120 years in eastern subpolar North Atlantic. Nature communications. 2020;11(1):585.

3. Houghton RW, Visbeck MH. Quasi-decadal salinity fluctuations in the Labrador Sea. Journal of Physical Oceanography. 2002;32(2):687-701.

4. NOAA National Centers for Environmental Information. World Ocean Atlas Climatology - Time period: Averaged decades Annual salinity objectively analyzed mean at the surface (1.00 degree grid);. Available from: `https://www.ncei.noaa.gov/access/world-ocean-atlas-2023f/bin/woa23f.pl?parameterOption=s`.

5. Cessi P. A simple box model of stochastically forced thermohaline flow. Journal of physical oceanography. 1994;24(9):1911-20.
