# Supplementary material for: Recovery-induced tipping in Stommel’s kicked ocean box model
Source: PLoS One. 2026 Feb 3;21(2):e0342171. doi: 10.1371/journal.pone.0342171 (PMC12867227; doi:10.1371/journal.pone.0342171)
Supplement: Attachment — Submitted filename: Reviewer comments_Plos One.pdf [file pone.0342171.s003.pdf]

Recovery-induced tipping in Stommel's kicked ocean box model

Manuscript Number: PONE-D-25-36894

Comments to the authors

General assessment

This manuscript presents a mathematically rigorous and conceptually novel investigation into the dynamics of "recovery-induced tipping" in Stommel's kicked ocean box model—a stylized representation of thermohaline ocean circulation. The authors expand upon prior work by analyzing the robustness of this tipping phenomenon across disturbance parameter space and identifying the bifurcation mechanisms driving the behavior. Furthermore, they explore an analogous behavior in a continuously perturbed variant of the model. The study is well-organized, clearly written, and supported by detailed computational simulations and bifurcation analyses. The manuscript makes a valuable contribution to theoretical climate dynamics and nonlinear system resilience by highlighting the counterintuitive result that longer recovery times can destabilize rather than stabilize a system. However, some areas require clarification, expansion, and editorial improvement to strengthen the manuscript's impact and accessibility.

Abstract

The abstract effectively summarizes the study's objectives and key findings. However, it may benefit from a clearer statement on the broader implications of the findings beyond conceptual model dynamics—particularly regarding the potential for analogous tipping in real oceanographic or climate systems. It may attract the general audience more.

Additionally, the authors should consider clarifying early that the model is conceptual and idealized, which will temper overinterpretation.

Introduction

The introduction is well-structured and provides sufficient background on flow-kick systems, bifurcation behaviour, and Stommel's two-box model. The motivation for studying "recovery-induced tipping" is well-articulated, but the potential applications to broader Earth system tipping phenomena could be more explicitly stated.

The authors may briefly introduce the ecological or climatological relevance of Stommel's model (e.g., connection to AMOC) upfront to better ground the reader.

Model

2.1 **Stommel's two-box model:** The authors provide a clear derivation of the nondimensionalized two-box system. However, the authors should emphasize the idealized assumptions behind the model, such as instantaneous mixing, spatial homogeneity, absence of vertical stratification or inertia. These assumptions should

be acknowledged explicitly to clarify limitations when drawing analogies to real-world AMOC dynamics.

2.2 Salinity disturbances via kicks and flows: The use of a flow-kick map to model salinity perturbations is mathematically sound and conceptually interesting. The interpretation of κ as representing freshwater pulses is appropriate, but the manuscript could benefit from a short paragraph highlighting how plausible such periodic "kicks" are in the real ocean. The authors should clarify the ecological or physical mechanisms by which these kicks might realistically occur (e.g., meltwater surges, extreme precipitation events).

Basin outcomes and circulation reversal

This section is central to the manuscript and well-developed. Figure 3a is informative but visually dense. Adding an inset zoom-in or improving the labelling (e.g., legend directly in the figure for hatched/dotted zones) would enhance clarity.

The mechanistic reasoning for why longer τ can push trajectories into the basin of C is briefly mentioned but underdeveloped in this section—it may help to provide a short schematic or intuitive explanation earlier (before Section 4).

The use of the separatrix, basin identification, and flow-kick outcomes is rigorous. However, the language used to describe "tipping" might be confusing to non-mathematical readers (like me). Consider stating explicitly that this refers to a switch in the long-term attractor (i.e., final state), not just temporary deviation.

The discussion of circulation reversal is important and clearly separated from basin tipping—this distinction is appreciated.

Continuation and bifurcation of flow-kick fixed points

This is a strong section and provides an excellent mechanistic explanation for the observed tipping behaviour using bifurcation theory.

It would be helpful to include a clarification on whether the Jacobians were calculated analytically or numerically, some information on tolerances or convergence criteria for Newton's method and a brief explanation of how fixed-point stability was confirmed (e.g., eigenvalue analysis). Adding a brief explanation on any assumptions or tolerance settings used. This is particularly relevant for readers wanting to reproduce the results.

It would be helpful to include quantitative bifurcation coordinates (e.g., $\tau \approx 4.9$, $\tau \approx 0.23$) in the figure caption and directly label the attractors in Fig 4b.

Clarify in the text how the global attractivity of $\hat{C}$ was confirmed—through simulation or stability analysis?

Including a short sentence about the relevance of these bifurcations to resilience and early warning in dynamical systems could enhance interdisciplinary appeal to a general audience.

Continuous salinity disturbances

This section nicely bridges discrete and continuous forcing approaches. It may help to briefly note here (rather than waiting until the Discussion) that physical systems rarely experience precisely constant or discrete perturbations—thus hybrid modelling frameworks might be more realistic. How does a steady freshwater flux at $r = 0.1$ compare in plausibility to a $\kappa = 0.1$ kick every 1 year?

It may help to explicitly summarize what is gained or lost in terms of realism or predictability when using the continuous vs. discrete model.

Clarify whether the Cr branch always remains stable, or whether any instabilities were observed.

Discussion

6.1 Dynamic implications of recovery-induced tipping: This is a valuable reflection, especially the concept that increased disturbance may enable escape from hysteresis. This inversion of classical resilience thinking is an important insight.

The authors may consider expanding on how this finding might influence real-world resilience strategies in climate, ecology, or engineered systems.

6.2 Model comparison to geophysical measurements: The attempt to ground model parameters in real oceanographic data is commendable and handled with caution. That said, the manuscript could further emphasize the conceptual nature of the comparison. Phrases like “possible but unlikely” or “idealized representation” could help avoid overinterpretation by non-specialist readers.

A table summarizing estimated κ and τ values for the different oceanographic events (GSAs, Greenland meltwater) could be useful.

6.3 Future directions: These are well-motivated. The idea of exploring stochasticity in flow times and kick magnitudes is especially promising. The authors may consider also suggesting potential for coupling this framework with data-driven or machine learning methods to identify or predict tipping thresholds in empirical datasets.
