# Supplementary material for: Recovery-induced tipping in Stommel’s kicked ocean box model
Source: PLoS One. 2026 Feb 3;21(2):e0342171. doi: 10.1371/journal.pone.0342171 (PMC12867227; doi:10.1371/journal.pone.0342171)
Supplement: Attachment — Submitted filename: Response to Reviewers.pdf [file pone.0342171.s004.pdf]

Authors' Response to Reviews of

Recovery-induced tipping in Stommel's kicked ocean box model

P. Kelly, X. Leng, P. Cogan, A. Jing, K. Meyer
PLOS ONE, PONE-D-25-36894

R[#]C: ***Reviewer [#] Comment***, AR: Authors' Response, ☐ Manuscript Text

General Comments

R2C: ***Response "No" to Question 3: Have the authors made all data underlying the findings in their manuscript fully available?***

AR: To our knowledge and by Reviewer One's judgement, we have made all data—in our case, code—underlying the findings in our manuscript fully available. We would be happy to follow up on a specific data request.

R2C: ***[T]he authors must improve the quality of the plots.***

AR: We have followed Reviewer One's suggestions to improve the clarity of Figure 3a by adding an inset with labels. We would be happy to respond to specific feedback regarding any of the plots.

Abstract

R1C: ***The abstract effectively summarizes the study's objectives and key findings. However, it may benefit from a clearer statement on the broader implications of the findings beyond conceptual model dynamics—particularly regarding the potential for analogous tipping in real oceanographic or climate systems. It may attract the general audience more.***

AR: We appreciate this suggestion. We have rephrased the end of the last sentence from "similar phenomena may occur in other models" to "similar phenomena may occur in other models and real-world systems," to emphasize the potential for real-world implications. We chose not to restrict the domain of possibility to climate or oceanography.

R1C: ***[T]he authors should consider clarifying early that the model is conceptual and idealized, which will temper overinterpretation.***

AR: Good point. We added the word "idealized" in "Stommel's idealized two-box model" to clarify this.

1. Introduction

R1C: ***The motivation for studying "recovery-induced tipping" is well-articulated, but the potential applications to broader Earth system tipping phenomena could be more explicitly stated.***

AR: Thank you for this suggestion. We have added the following at lines 40–45:

To this list we add recovery-induced tipping, both in the forward direction—in which lengthening the recovery period between disturbances can trigger a shift to an alternative attractor—and in the backward direction—in which increasing disturbance frequency can trigger a shift back. This latter possibility, discussed in detail in Section 6.1, may be relevant in Earth systems as climate change increases the frequencies of disturbances such as hurricanes and wildfires.

R2C: ***The authors must also include recent citations in the introduction part of the manuscript.***

AR: We have added a citation at line 40 to a 2012 article that is central in the tipping literature:

> Processes known to trigger tipping in climate models include bifurcations, noise, and quickly changing parameters (rate-induced tipping) (see [1] and references therein).

We also added a reference to a modern review article of climate tipping points at line 4:

> Stommel's two-box model ... laid a theoretical foundation for scientists to study tipping between alternative stable regimes in the Atlantic Meridional Overturning Circulation (AMOC) [2] ...

R1C: ***The authors may briefly introduce the ecological or climatological relevance of Stommel's model (e.g., connection to AMOC) upfront to better ground the reader.***

AR: To orient the reader to Stommel's model and its relevance to climate, we added this brief paragraph at the beginning of the introduction (lines 2–7):

> Stommel's two-box model of thermohaline oceanic circulation [3] laid a theoretical foundation for scientists to study tipping between alternative stable regimes in the Atlantic Meridional Overturning Circulation (AMOC) [2], a climate subsystem that transfers heat and salt from low to high latitudes. In this paper we incorporate pulses of freshwater into Stommel's model as a case study for tipping via non-intuitive flow-kick dynamics.

2. Model

R1C: ***The authors provide a clear derivation of the nondimensionalized two-box system. However, the authors should emphasize the idealized assumptions behind the model, such as instantaneous mixing, spatial homogeneity, absence of vertical stratification or inertia. These assumptions should be acknowledged explicitly to clarify limitations when drawing analogies to real-world AMOC dynamics.***

AR: Thank you for this suggestion. We have added the following at lines 75–77:

> The circulation q through the bottom capillary tube and surface channel is driven by density differences between the fluids in the boxes, which are in turn determined by temperature and salinity (see Eq 2). Importantly, the model assumes instantaneous mixing within each box, so does not resolve spatial variation within the boxes such as vertical stratification. The model also ignores inertia associated with circulation.

R1C: ***The interpretation of κ as representing freshwater pulses is appropriate, but the manuscript could benefit from a short paragraph highlighting how plausible such periodic "kicks" are in the real ocean. The***

authors should clarify the ecological or physical mechanisms by which these kicks might realistically occur (e.g., meltwater surges, extreme precipitation events).

AR: We agree this information is needed when introducing kicks. We have added the following short paragraph in Section 2.2 (lines 123–128):

> Kicks are a stylized model of recurrent salinity perturbations in the real ocean. For example, Greenland contributes annual pulses of freshwater flux to the North Atlantic in the summer months, in addition to baseline flow [4]. Kicks represent the simplifying assumption that all water associated with a pulse arrives instantaneously in time. See Section 6.2 for further comparison between the kicked ocean box model and geophysical processes.

3. Basin outcomes and circulation reversal

R1C: ***Figure 3a is informative but visually dense. Adding an inset zoom-in or improving the labelling (e.g., legend directly in the figure for hatched/dotted zones) would enhance clarity.***

AR: Thank you for these ideas to make the figure more digestible. We have added a zoomed-in inset in Figure 3a for $0 \leq \kappa \leq 0.2$ and labelled circulation directions and other features directly within the inset.

R1C: ***The mechanistic reasoning for why longer τ can push trajectories into the basin of C is briefly mentioned but underdeveloped in this section—it may help to provide a short schematic or intuitive explanation earlier (before Section 4).***

AR: Thank you for this suggestion. While we have chosen to organize the manuscript by separating the description of model behavior in Section 3 from the analysis of why it happens in Section 4, we have added a sentence at lines 147–149 in Section 3 to alert the reader where to find the explanation:

> Section 4 analyzes the mechanism underlying this recovery-induced tipping: a saddle-node bifurcation coupled with a stable flow-kick fixed point migrating across the separatrix between the basins of A and C.

R1C: ***The use of the separatrix, basin identification, and flow-kick outcomes is rigorous. However, the language used to describe "tipping" might be confusing to nonmathematical readers (like me). Consider stating explicitly that this refers to a switch in the long-term attractor (i.e., final state), not just temporary deviation.***

AR: To clarify the meaning of "tipping" for a broader audience, we have added the following to the introduction (lines 37–40), after first using the word "tip":

> Tipping in dynamical systems involves a change in the long-term fate of a trajectory towards a different attractor. Processes known to trigger tipping in climate models include bifurcations, noise, and quickly changing parameters (rate-induced tipping) (see [1] and references therein).

4. Continuation and bifurcation of flow-kick fixed points

R1C: ***It would be helpful to include a clarification on whether the Jacobians were calculated analytically or numerically, some information on tolerances or convergence criteria for Newton's method and a***

brief explanation of how fixed-point stability was confirmed (e.g., eigenvalue analysis). Adding a brief explanation on any assumptions or tolerance settings used. This is particularly relevant for readers wanting to reproduce the results.

AR: We have added the following details regarding methods for finding fixed points and stabilities (lines 202–208):

> Flow-kick fixed points were found by applying Newton's method with an error tolerance of 10^{-10} to the function
>
> $$\vec{F}(x,y) = \vec{G}_{\tau,\kappa}(x,y) - \begin{bmatrix} x \\ y \end{bmatrix}$$
>
> using the MATLAB script `Fig4a.m`, which calls functions `Newton_2d.m` and `CoupledVar_2d.m`. The latter function computes Jacobian matrices for Newton's method numerically using the variational equation for the spatial derivative of a flow. Stability of flow-kick fixed points was inferred from behavior of nearby flow-kick trajectories.

R1C: ***It would be helpful to include quantitative bifurcation coordinates (e.g., $\tau \approx 4.9$, $\tau \approx 0.23$) in the figure caption and directly label the attractors in Fig 4b.***

AR: We have added stability information and (x, y) bifurcation coordinates to the caption below line 208 as follows:

> (b) As τ decreases from 9, the branches of fixed points maintain their stability, but $\widehat{A}_\tau$ and $\widehat{B}_\tau$ undergo a saddle-node bifurcation at $\tau = 4.9$ and $(x, y) \approx (0.26, 0.51)$, while the branch $\widehat{C}$ persists and crosses into the original basin of A at $\tau = 0.23$ and $(x, y) \approx (0.41, 0.41)$.

We chose not to add additional labels to Figure 4b regarding stability in order to avoid overcrowding the plot.

R1C: ***Clarify in the text how the global attractivity of $\hat{C}$ was confirmed—through simulation or stability analysis?***

AR: We had noted the global stability of $\hat{C}$ through simulation in a rather ad-hoc manner, and this comment prompted us to study it methodically using a new MATLAB script. We discovered multiple stable flow-kick fixed points for a small slice of (τ, κ) values, and so we modified our discussion of global stability at lines 212–219 as follows.

> We expect that below the dashed curve, the qualitative picture resembles Fig 4a, with three flow-kick fixed points. Above the dashed curve, and for $0 < \kappa \leq 0.4$, numerical simulations using the script `FKGlobalStability.m` suggest the existence of a globally attracting flow-kick fixed point $\widehat{C}$.
>
> The different basin outcomes above the dashed saddle-node bifurcation curve in Fig 3a stem from different positions of the [DELETED: globally] remaining attracting flow-kick equilibrium or equilibria [DELETED: $\widehat{C}$].

R1C: ***Including a short sentence about the relevance of these bifurcations to resilience and early warning in dynamical systems could enhance interdisciplinary appeal to a general audience.***

AR: We agree that making these connections enhances the interdisciplinary appeal of the article. Because we devote a subsection of the Discussion to this topic, we have included a sentence at lines 229–230 that points readers to this section:

See Section 6.1 for discussion of the broader relevance of recovery-induced tipping to resilience in dynamical systems.

5. Continuous salinity disturbances

R1C: ***This section nicely bridges discrete and continuous forcing approaches. It may help to briefly note here (rather than waiting until the Discussion) that physical systems rarely experience precisely constant or discrete perturbations—thus hybrid modelling frameworks might be more realistic. How does a steady freshwater flux at $r = 0.1$ compare in plausibility to a $\kappa = 0.1$ kick every 1 year? It may help to explicitly summarize what is gained or lost in terms of realism or predictability when using the continuous vs. discrete model.***

We have added a sentence that highlights the first point at the beginning of Section 5:

> Kicks are an idealized representation of freshwater inputs to the North Atlantic, which appear in datasets as continuous but peaked discharge curves (e.g. [5, 4]).

The second question regarding predictability based on continuous versus discrete models strikes us as an interesting and non-trivial research question that is outside the scope of the present study.

R1C: ***Clarify whether the Cr branch always remains stable, or whether any instabilities were observed.***

AR: We have added the following details regarding the stability of the C_r branch:

> Numerical simulations indicate that once r exceeds ≈ 0.028, the branch C_r of equilibria emanating from C is not only locally stable but attracts all trajectories within $[0, 1] \times [0, 1]$ (see `CtsGlobalStability.m`).

6. Discussion

R1C: ***Dynamic implications of recovery-induced tipping: This is a valuable reflection, especially the concept that increased disturbance may enable escape from hysteresis. This inversion of classical resilience thinking is an important insight. The authors may consider expanding on how this finding might influence real-world resilience strategies in climate, ecology, or engineered systems.***

To expand on these ideas, we have added the following at lines 319–325:

> We expect this behavior more generally in systems in which intensifying disturbance eliminates one attractor and drives an alternative attractor into its original basin. Intensifying and then ceasing a disturbance to ultimately undo its effects would be a counterintuitive strategy for managing an ecosystem or climate subsystem. But perhaps this strategy may be relevant in some situations. While these authors do not endorse large-scale experimentation, the phenomenon certainly warrants further study.

R1C: ***Model comparison to geophysical measurements: The attempt to ground model parameters in real oceanographic data is commendable and handled with caution. That said, the manuscript could further***

emphasize the conceptual nature of the comparison. Phrases like "possible but unlikely" or "idealized representation" could help avoid overinterpretation by non-specialist readers.

To emphasize this important point, we have added the purple text at lines 350-351:

> Although any quantitative comparisons between Stommel's idealized model and real-world data should be treated with caution, we note that a weakening temperature-dominated circulation regime in Stommel's model is consistent with modern observations of the AMOC [6].

and at line 370:

> Thus a (τ, κ) disturbance regime modeled after Greenland inputs again lies in region (i) of Figure 3a, and is likely far away from any recovery-induced tipping threshold.

R1C: ***A table summarizing estimated κ and τ values for the different oceanographic events (GSAs, Greenland meltwater) could be useful.***

We have added such a table at line 340.

R1C: ***Future directions: These are well-motivated. The idea of exploring stochasticity in $flow$ times and kick magnitudes is especially promising. The authors may consider also suggesting potential for coupling this framework with data-driven or machine learning methods to identify or predict tipping thresholds in empirical datasets.***

AR: We appreciate this suggestion, but have decided to keep the scope of our suggested future directions within the realm of our expertise.

References

[1] Peter Ashwin, Sebastian Wieczorek, Renato Vitolo, and Peter Cox. Tipping points in open systems: bifurcation, noise-induced and rate-dependent examples in the climate system. *Philosophical Transactions of the Royal Society A: Mathematical, Physical and Engineering Sciences*, 370(1962):1166–1184, 2012.

[2] David I Armstrong McKay. Two decades of climate tipping points research: Progress and outlook. *Dialogues on Climate Change*, 1(1):30–38, 2024.

[3] Henry Stommel. Thermohaline convection with two stable regimes of flow. *Tellus*, 13(2):224–230, 1961.

[4] Jonathan Bamber, Michiel Van Den Broeke, Janneke Ettema, Jan Lenaerts, and Eric Rignot. Recent large increases in freshwater fluxes from greenland into the north atlantic. *Geophysical Research Letters*, 39(19), 2012.

[5] Andreas P Ahlstrøm, Dorthe Petersen, Peter L Langen, Michele Citterio, and Jason E Box. Abrupt shift in the observed runoff from the southwestern greenland ice sheet. *Science Advances*, 3(12):e1701169, 2017.

[6] Levke Caesar, Stefan Rahmstorf, Alexander Robinson, Georg Feulner, and V Saba. Observed fingerprint of a weakening atlantic ocean overturning circulation. *Nature*, 556(7700):191–196, 2018.
